# Chronic exercise reduces hypothalamic transforming growth factor-β1 in middle-aged obese mice

**DOI:** 10.18632/aging.101281

**Published:** 2017-08-28

**Authors:** Vagner R. R. Silva, Carlos K. Katashima, Luciene Lenhare, Carla G. B. Silva, Joseane Morari, Rafael L. Camargo, Licio A. Velloso, Mario A. Saad, Adelino S. R. da Silva, Jose Rodrigo Pauli, Eduardo Rochete Ropelle

**Affiliations:** ^1^ School of Applied Sciences, University of Campinas, Limeira, SP, Brazil; ^2^ Department of Internal Medicine, University of Campinas, Campinas, SP, Brazil; ^3^ CEPECE - Research Center of Sport Sciences, School of Applied Sciences, University of Campinas, Limeira, SP, Brazil; ^4^ Laboratory of Cell Signaling, Obesity and Comorbidities Research Center, University of Campinas, Campinas, 1308-970, Brazil; ^5^ Postgraduate Program in Rehabilitation and Functional Performance, Ribeirão Preto Medical School, USP, Ribeirão Preto, SP, Brazil; School of Physical Education and Sport of Ribeirão Preto, University of São Paulo, Ribeirão Preto, SP, Brazil

**Keywords:** aging, obesity, hypothalamus, TGFβ-1, exercise

## Abstract

Obesity and aging are associated with hypothalamic inflammation, hyperphagia and abnormalities in the thermogenesis control. It has been demonstrated that the association between aging and obesity induces hypothalamic inflammation and metabolic disorders, at least in part, through the atypical hypothalamic transforming growth factor-β (TGF-β1). Physical exercise has been used to modulate several metabolic parameters. Thus, the aim of this study was to evaluate the impact of chronic exercise on TGF-β1 expression in the hypothalamus of Middle-Aged mice submitted to a one year of high-fat diet (HFD) treatment. We observed that long-term of HFD-feeding induced hypothalamic TGF-β1 accumulation, potentiated the hypothalamic inflammation, body weight gain and defective thermogenesis of Middle-Aged mice when compared to Middle-Aged animals fed on chow diet. As expected, chronic exercise induced negative energy balance, reduced food consumption and increasing the energy expenditure, which promotes body weight loss. Interestingly, exercise training reduced the TGF-β1 expression and IkB-α ser32 phosphorylation in the hypothalamus of Middle-Aged obese mice. Taken together our study demonstrated that chronic exercise suppressed the TGF-β1/IkB-α axis in the hypothalamus and improved the energy homeostasis in an animal model of obesity-associated to aging.

## INTRODUCTION

The aging is a natural process described such a loss of cellular functions, tissues and organs. Studies have shown that aging is a multifactorial phenomenon, but the lifestyle such as, sedentarism and poor diet habits, plays a critical role for lifespan [1]. Currently, the aging population has been increased together with sedentary lifestyle and high-caloric diet consumption, which contributed to the increase of obesity in aged people [2, 3]. The obesity in the older adult is largely related with cancer risk [2] and other age-related diseases [3, 4].

The hypothalamus controls energy balance and multiple metabolic signals including food intake and energy expenditure by neurotransmitters such as proopio-melanocortin (POMC) and neuropeptide Y (NPY) [5, 6]. However, aging and obesity are associated with hypothalamic inflammation and loss of anorexigenic and thermogenic signals in the hypothalamus [6, 7]. Hypothalamic inflammation is associated with several age-related diseases such as Alzheimer's disease, obesity, diabetes type 2, cancer, sarcopenia, with significant risks of morbidity and/or mortality [8]. In this scenario, the transforming growth factor-β (TGF-β), a member of the family of pleiotropic cytokines, has been associated with hypothalamic inflammation and metabolic disorders in obese and aging mice [9].

The TGF-β protein has three different isoforms (1-3), located in the extracellular matrix and can regulate several biological cell functions such as proliferation, differentiation, migration, and survival, playing pivotal role in the morphogenesis and homeostasis [10]. This protein is expressed in different tissues including brain [9], tumor [11], corneal epithelium [12] and muscle [13]. There are some evidences that links the TGF-β1 levels with obesity and aging in rodents and humans [9, 14, 15]. In addition, the TGF-β1 levels were correlated with adiposity in rodents and humans [14, 16]. Interestingly, the systemic treatment with anti-TGF-β1 antibody protected leptin-deficient (*ob/ob)* and diet-induced obese (DIO) mice against obesity and diabetes [14].

Recently, TGF-β1 was found to be highly expressed in the hypothalamus of obese and old mice [9]. Yan and colleagues showed that hypothalamic injection of TGF-β1 caused hyperglycemia and glucose intolerance in mice, conversely, the TGFβ-1 deficient mice (*Tgfb1^+/−^*) were protected from hypothalamic inflammation and type 2 diabetes induced by high-fat diet (HFD) [9]. The authors also demonstrated that TGF-β1 induced a hypothalamic RNA stress response, accelerating mRNA down regulation of IkBα that has the function of modulating negatively proinflammatory nuclear factor-κB [9]. Furthermore, the combination of poor diet consumption associated with a sedentary lifestyle during aging process may potentiate hypothalamic inflammation, stimulating central TGF-β1 expression, contributing to the metabolic syndrome, age-related disorders reducing the lifespan [6, 15]. Together, these evidences indicate that hypothalamic TGF-β1 could be considered an interesting target to control energy homeostasis, nevertheless, the mechanisms by which TGF-β1 protein levels are controlled in the hypo-thalamus remain unclear.

In the other hand, regular exercise can protect against several diseases and contribute to health maintenance [17]. Studies have shown that some peripheral [13, 18-20] and central [21] benefits of exercise are associated with the reduction of TGF-β1signaling, however, the effects of physical exercise on the hypothalamic TGF-β1 protein content are unknown. Thus, the aim of this study was to determine the influence of chronic exercise on hypothalamic TGF-β1 in middle-aged obese mice.

## RESULTS

### Effects of long-term of high-fat diet consumption in Middle-Aged mice

**Figure 1:**
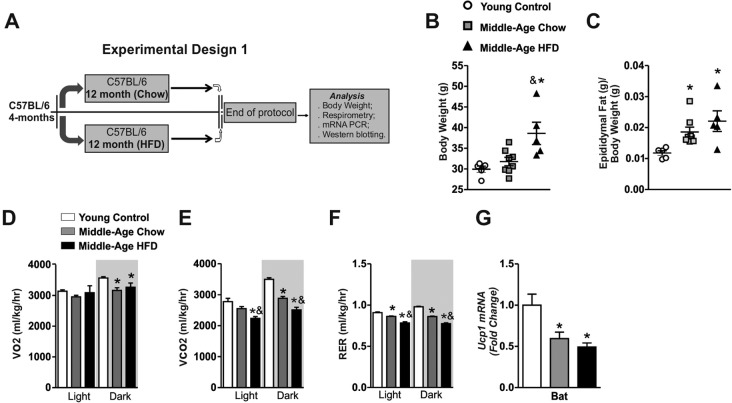
Effects of long-term of high-fat diet consumption in Middle-Age mice Experimental design 1 (**A**). Body weight and epididymal fat (**B** and **C**) (n=5-8 per group). VO_2_ (**D**), CO_2_ (**E**), RER (**F**) (n= 4-5 per group). *Ucp1* mRNA in the brown adipose tissue (n=5-8 per group) (**G**). The animals were fasted for 8 hours before the brown adipose tissue extraction. Data are expressed as means ± SEM. *, *p*<0.05 vs Young control group and ^&^, *p*<0.05 vs Middle-Age fed on chow diet.

Initially, we evaluated the effects of combination between aging and long-term of high-fat diet (HFD) treatment (twelve months) on energy homeostasis in mice. Body weight, energy expenditure, brown adipose tissue and hypothalamic samples were analyzed, as shown in the experimental design Fig. 1A.

The Middle-Aged mice (16-months old) fed on chow diet presented only a discrete body weight gain, but significant epididymal fat pad gain when compared to young control group (4-months old) (Fig. 1B and C). Also, these mice displayed lower values of VO_2_ consumption, CO_2_ production and respiratory exchange ratio (RER) in the dark period when compared to young control group (Fig. 1D-F). Consistent with these findings, Middle-Aged mice presented low levels of *Ucp1* mRNA in BAT (Fig.1G).

Thereafter, we evaluated the effects of HFD treatment in Middle-Aged mice. The long-term of HFD treatment increased body weight and epididymal fat pad mass gain when compared to other groups (Fig. 1B and C). The HFD consumption affected the CO_2_ production and respiratory exchange ratio (RER) in the dark period when compared to young and Middle-Aged mice fed on chow diet (Fig. 1E and F). The *Ucp1* mRNA levels in the brown adipose tissue were reduced in the Middle-Aged mice after HFD treatment when compared to young control group, but no difference was observed when compared to Middle-Aged mice fed on chow diet (Fig. 1G). Overall, these results suggest that long term of HFD treatment potentiates the abnormalities in the control of body weight gain and the thermogenesis of Middle-Aged mice.

### Long-term of HFD treatment increased hypothalamic TGF-β1 accumulation and inflammatory genes in Middle-Aged mice

**Figure 2:**
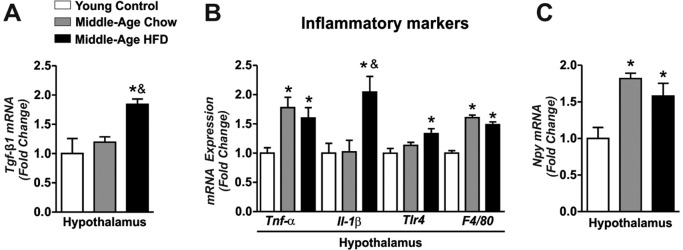
Effects of long-term of HFD on hypothalamic TGF-β1 accumulation and inflammatory genes in Middle-Age mice Real time PCR assay of hypothalamic *Tgf-β*1 (**A**), *Tnf-α, Il1-β, Tlr4, F4/80* (**B**) and *Npy* mRNA level (**C**) (n=4-7 per group). The animals were fasted for 8 hours before the hypothalamus extractions. Data are expressed as means ± SEM. *, *p*<0.05 vs Young control group and &, *p*<0.05 vs Middle-Age fed on chow diet.

Next, we observed slight augment of the Tgf-β-1 mRNA levels in the hypothalamus of Middle-Aged mice when compared to young control group, but no statistical difference was found (Fig. 2A). However, the long-term of HFD treatment markedly increased the Tgf-β-1 mRNA levels in the hypothalamus when compared to other groups (Fig. 2A). This phenomenon was accompanied by high levels of inflammatory markers Tumor Necrosis Factor Alpha (Tnf-α), Interleukin-1- Beta (*Il1-β)*, Toll-Like- Receptor 4 (*Tlr4)*, and *F4/80* mRNA levels in the hypothalamic samples of Middle-Aged mice fed on HFD when compared to young control and Middle-Aged mice fed on chow diet (Fig. 2B). Both, aging and HFD increased the *Npy* mRNA levels in the hypothalamus when compared to young control group (Fig. 2C).

We also monitored the *Tgf-β1* mRNA levels in the BAT. Interestingly, *Tgf-β1* mRNA levels were reduced in Middle-Aged groups when compared to young control group (Fig. S1A). Thus, these results suggest that HFD consumption potentiates the hypothalamic TGF-β1 expression and the inflammatory profile in Middle-Aged mice.

### Chronic exercise reduces body weight and restores thermogenesis in Middle-Aged obese mice

**Figure 3:**
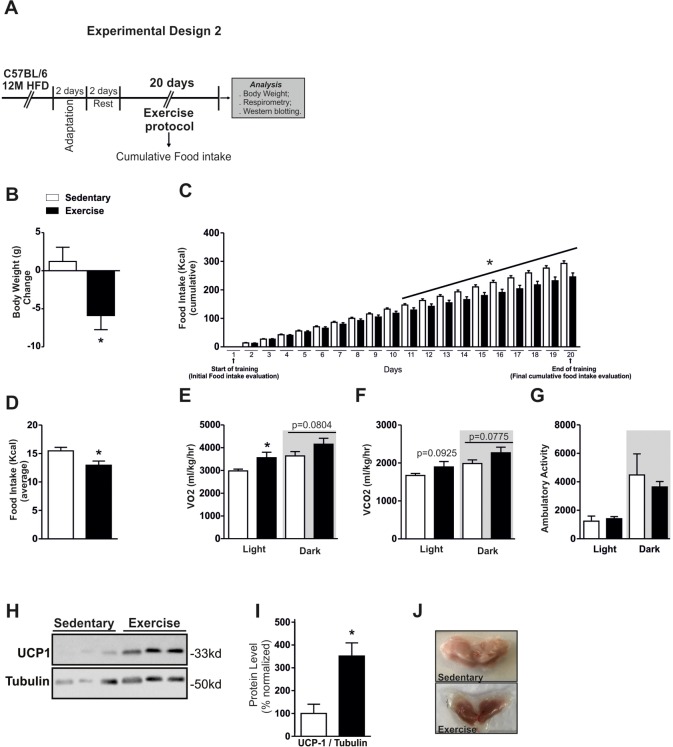
Effects of chronic exercise in Middle-Aged obese mice Experimental design 2 (**A**). Body weight change, cumulative food intake and average food intake (**A-D**) (n=10 per group). VO_2_ (**E**), CO_2_ (**F**) and ambulatory activity (**G**) (n= 4 per group). All analyses were made in the last day of training. Western blotting of UCP-1 protein level in BAT (**H**) and normalization of protein level by Tubulin (right) (**I**), picture of BAT, highlighting the coloration (**J**) (n= 6 per group). Data are expressed as means ± SEM. *, *p*<0.05 vs Sedentary group.

After these preliminary results, we hypothesized that the physical exercise could be an interesting physiological stimulus for modulating the hypothalamic TGF-β1 levels in our experimental model. Thus, we performed an experiment to analyze the effects of chronic exercise only in the Middle-Aged obese mice, as presented in the experimental design in Fig. 3A.

We first evaluated some physiological parameters in response to the exercise training. Chronic exercise reduced the body weight and cumulative food in Middle-Aged obese mice when compared to the Middle-Aged obese sedentary group (Fig. 3B and C). We observed that cumulative food consumption day by day started to reduce significantly from the eleventh day of training (Fig. 3C). The average of food intake during the experiment was also lower in the exercised group (Fig. 3D). The VO_2_ consumption was increased in the light cycle in exercised group (Fig. 3E). A slight augment on VO_2_ consumption and CO_2_ production in the dark period was observed in the exercised Middle-Aged obese mice when compared to the Middle-Aged obese sedentary group, but no statistical differences were detected (Fig. 3E and F). No difference was found in the ambulatory activity between the groups (Fig. 3G). The analysis of ambulatory activity showed differences in just one point at the light cycle (Fig. S2A). Although the exercise increased modestly the VO_2_ consumption and CO_2_ production, the Western blot analysis demonstrated a significant augment of UCP1 protein content in BAT (Fig. 3H and I). Consistent with these data, we observed a visual changing in the coloration of brown adipose tissue (Fig. 3J). These results demonstrated that the chronic exercise-induced a negative energy balance, reducing the food consumption and increasing the energy expenditure in Middle-Aged obese mice.

### Chronic exercise reduces TGF-β1 levels and suppresses the inflammatory signaling in the hypothalamus of Middle-Aged obese mice

Next, we examined whether chronic exercise could regulate the hypothalamic TGF-β1 pathway. This phenomenon could be important once TGF-β1 induces hypothalamic RNA stress response that activates NFkB signaling and accelerates down-regulation of IkBα and proinflammatory pathways [9]. Thus, beyond the TGF-β1 we also investigated the hypothalamic IκB-α phosphorylation in Middle-Aged obese mice.

**Figure 4:**
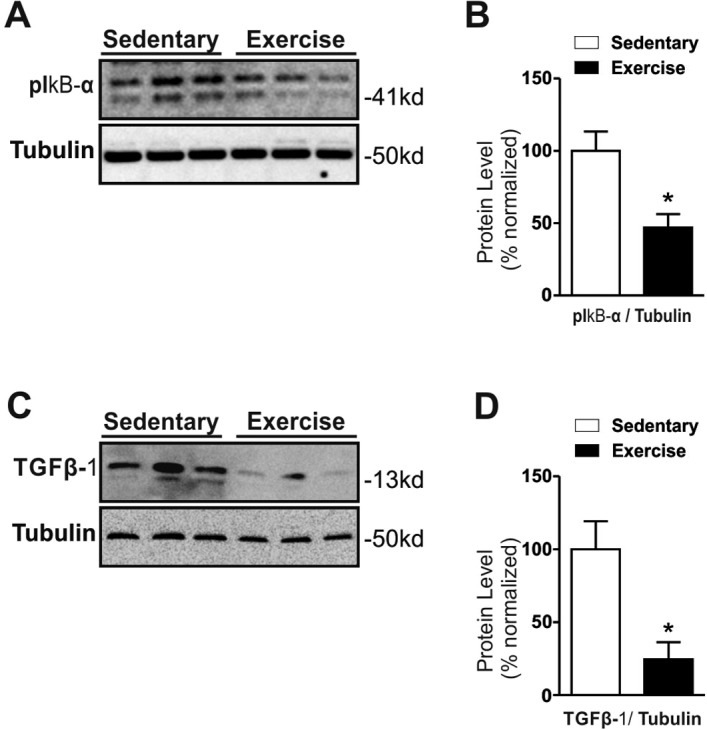
Effects of chronic exercise on hypothalamic TGF-β1 protein levels in Middle-Aged obese mice Western blotting of IkB-α ser32 phosphorylation (**A**) and TGF-β1 protein level (**C**) and Protein level normalization with Tubulin (right) (**B** and **D**) (n= 8 per group). All analyses were made after the last day of training. Data are expressed as means ± SEM. *, *p*<0.05 vs Sedentary group.

The chronic exercise reduced the hypothalamic IκB-α phosphorylation when compared to the Middle-Aged obese sedentary group (Fig. 4A and B). This data was accompanied by a strong reduction of TGF-β1 protein levels in exercised mice (Fig. 4C and D). Finally, we also analyzed the serum and hypothalamic levels of IL-6, but no difference was found in these parameters (*p=0.3066 for serum level* and *p=0.1960 for hypothalamus)* (Fig. S2B and C). Collectively, our results suggest that the chronic exercise can help to control hypothalamic TGF-β1/IκB-α axis in Middle-Aged obese mice.

## DISCUSSION

The obesity in elderly people is correlated with cancer and other age-related diseases [2-4]. Aging can affect several physiological functions during the life. Some of these metabolic and physiological dysfunctions are intimately associated with inflammatory responses that can reduce lifespan. The hypothalamic inflammation is associated with several metabolic abnormalities, which reduces lifespan [6, 22]. Conversely, regular exercise can improve several metabolic parameters, reversing or attenuating chronic diseases [23]. Here, we report that mice that consumed HFD during the aging process had high TGF-β1 protein levels and increased hypothalamic inflammatory responses, which regulated negatively their energy homeostasis. However, chronic exercise reduced the TGF-β1 protein levels and attenuated the inflammatory signaling in the hypothalamus, modulating the body weight and energy expenditure in Middle-Aged obese mice.

Studies have shown that TGF-β1, a family of pleiotropic cytokines, is associated with the development of obesity, diabetes type 2, cancer anorexia and aging [9, 11, 14, 15]. Lin et al. showed that the serum levels of TGF-β1 have a positive correlation with age, lifestyle, cigarette smoking and alcohol drinking in humans [15]. In addition, it has been reported that TGF-β1 serum levels are increased in *ob/ob* mice, HFD fed mice and obese humans [14, 15]. In the present study, we observed that sedentary mice that consumed HFD during the long part of their life acquired an obese phenotype and displayed strong increases of hypothalamic TGF-β1 protein levels. These results are in accordance with other study showing that obesity and aging increased hypothalamic TGF-β1 levels [9]. In our study, we observed that, at least, in 16-months-old mice fed on chow diet, the age did not affect significantly the hypothalamic TGF-β1 mRNA levels. Probably, the hypothalamic TGF 1βaccumulation begins to increase in Middle Age and reaches higher concentrations in older animals, however, the chronic HFD feeding accelerates this process in Middle-Age animals.

The long-term of HFD treatment strongly modulated classical inflammatory markers such as *Tnf-α*, *Il1-β*, *Tlr4* and *F4/80* mRNA levels in the hypothalamus of Middle-Aged animals. These inflammatory markers can negatively regulate the energy homeostasis, linking some age- and obesity-related metabolic disorders [24-28]. The central NPY signaling controls food intake and energy expenditure in mammals and plays an important role in the control of energy homeostasis [6]. Here, we found that independent of the type of the diet, the *Npy* mRNA levels were increased in Middle-Aged sedentary mice. However, the HFD feeding did not promoted an additional effect on NPY mRNA levels in Middle-Age mice. Besides that, it has been shown that the Npy mRNA levels increase after 2 weeks and decrease after 12 weeks of HFD treatment [29, 30], demonstrating that chronic exposure to HFD induces NPY mRNA stabilization in the hypothalamus and other mechanisms cold be involved in the body weight gain during the long-term of HFD treatment.

The TGF-β may have pro- or anti-inflammatory effects in different tissues, however, the mechanism for this regulation remains unclear [31, 32]. Rao *et al* showed that meteorin-like 1 (Metrnl), a circulating factor produced by muscle contraction after exercise and in adipose tissue in low temperatures, increased *Ucp1* mRNA expression, which was accompanied by an increase of the anti-inflammatory genes *Il-10* and *Tgf-β* mRNA levels in BAT [31]. Yadav and colleagues showed that an intraperitoneal injection of TGF-β1 reduced the UCP1 protein levels in lean mice [14]. Here, we found that *Tgf-β1* mRNA levels were decreased in BAT of Middle-Aged fed on HFD, which was accompanied by a reduction of *Ucp1* mRNA levels as described by Rao et al [31]. Although, we consider the possibility that TGF-β1 can exert an anti-inflammatory role in BAT, further studies are necessary to explain this duality.

It has been demonstrated that TGF-β1 levels are correlated the with body mass index (BMI), fat mass, and VO_2_ consumption in humans. In addition, high TGF-β1 levels were associated with atypical metabolic profile [14]. In this study, we detected a reduced VO_2_ consumption, CO_2_ production, and RER in Middle-Aged mice with higher hypothalamic TGF-β1 levels. The strong reduction in the RER of the Middle-Aged fed on HFD indicates that this group could use more fat as an energy source [33] or develops an energy expenditure dysfunction. To evaluate the energy expenditure, we also monitored the UCP1 in the brown adipose tissue. It is important to point out that BAT dysfunction may play important roles in some metabolic disorders such as obesity, diabetes, and cardiovascular disease [34], as well as during the aging process [35, 36]. We found that *Ucp1* mRNA levels were decreased in the BAT of Middle-Aged groups, without additional effects after the long-term of HFD treatment.

Regular exercise has the ability to improve thermogenic capacity by the activation of proteins in the brown adipose tissue such as UCP1, protecting against body fat accumulation and other metabolic diseases [37]. We verified an increase in the energy expenditure and an overexpression of UCP1 (∼200%) in Middle-Aged obese mice after the chronic exercise. Also, we observed an intensification of reddish coloration of BAT in the exercised animals when compared to the sedentary group. These data suggest that both the number of mitochondria and thermogenic capacity of BAT were increased [38]. These findings are interesting and show that regular exercise modulated the energy expenditure in Middle-Aged obese mice.

It has been demonstrated that exercise modulates several inflammatory and anti-inflammatory markers in the body. For instance, the physical exercise stimulates the IL-6, a cytokine, describe as inflammatory and anti-inflammatory molecule [39, 40]. We previously reported that acutely exercise suppressed the energy intake in obese rodents via IL-6 central action [39]. In the present study, we monitored the peripheral and central levels of IL-6 and we confirmed that IL-6 was not involved in the reduction of food consumption during the chronic exercise.

Strategies to control peripheral and central TGF-β1 signalling may be an innovative strategy for preventing metabolic and inflammatory diseases as well as age-related disorders. Therefore, we used chronic exercise, a non-pharmacologic and non-invasive strategy, for the control of diseases, as previously described [17]. Kim and Lee demonstrated that twenty-four days of swimming exercise was able to decrease TGF-β1 levels in vascular fibrosis in aged obese rats [41]. Böhm and collaborators showed that eight weeks of training reduced the TGF-β levels in skeletal muscle in twenty Middle-Aged subjects, while muscular TGF-β expression was associated with the decline of mitochondrial oxidation and insulin sensitivity [13]. Similar results were found by Pincu et al. [18] and Touvra et al. [19]. Thus, for the first time, we showed that moderate chronic exercise reduced the TGF-β1 protein levels (∼75%) and IκB-α phosphorylation (∼50%) in the hypothalamus of Middle-Aged obese mice. Interestingly, these data were accompanied by a reduction of food consumption and body weight.

In conclusion, we identified that HFD-feeding during the aging process induced TGF-β1 accumulation and inflammatory genes in the hypothalamus of Middle-Aged obese mice. These data were accompanied by body weight gain and modification in the energy expenditure pattern. However, chronic exercise reduced the TGF-β1 protein levels and the inflammatory signalling. In parallel, exercise protected against obesity, reducing the food consumption and increasing thermogenesis. Taken together, these data suggest that regular physical exercise can control energy homeostasis at least in part, through the reduction of the hypothalamic TGF-β1/IκB-α axis in Middle-Aged obese mice.

## MATERIALS AND METHODS

### Animals and diet

Four-months-old male C57BL/6J mice were obtained from the University of Campinas Breeding Center (CEMIB). The animals were randomly separated into two groups: One group consumed standard chow diet (Chow) (3.948 kcal·kg^−1^), while the other group consumed high-fat diet (HFD) (5.358 kcal·kg^−1^) *ad libitum* for twelve months. Four-months-old C57BL/6J mice were used as a control group. (see Table 1 and experimental design 1 Figure 1A). Next, part of these mice that consumed HFD for twelve months were submitted to the chronic swimming exercise protocol (Exercise group), and the respective control group was maintained sedentary (Sedentary group). During this experimental period, these animals were maintained in a 12:12 hour light and dark cycle and housed in cages between 22–24°C with free access to food and water. The light cycle started at 6:00 am. The experiments were approved by the ethics committee of the University of Campinas (number:2736-1), which follows the international guidelines for the use of animals in experimental studies and experiments.

**Table 1:** Components of standard chow diet and high-fat diet

Ingredients	Standard chow		High-fat diet	
	g kg^−1^	kcal kg^−1^	g kg^−1^	kcal kg^−1^
Cornstarch (Q.S.P.)	397.5	1590	115.5	462
Casein	200	800	200	800
Sucrose	100	400	100	400
Dextrinated starch	132	528	132	528
Lard	-	-	312	2808
Soybean Oil	70	630	40	360
Cellulose	50	-	50	-
Mineral Mix	35	-	35	-
Vitamin Mix	10	-	10	-
L-Cystine	3	-	3	-
Choline	2.5	-	2.5	-
Total	1000	3948	1000	5358

Consume standard chow diet (Chow) (3.948 kcal·kg-1) and high-fat diet (HFD) (5.358 kcal·kg-1) ad libitum for twelve months.

### Chronic exercise protocol

Mice were acclimated to swimming for two days, ten minutes per day. Water temperature was maintained at 32°C. The mice swam in groups of four in plastic barrels of 40 cm in diameter that were filled to a depth of 20 cm for one hour, during five days per twenty days. Extractions of tissue were performed after the last session of the exercise protocol.

### Food consumption

Cumulative food consumption and body weight measurements were monitored every day during the exercise protocol in individual cages.

### Brown adipose tissue (BAT) photos

Images were taken immediately after extraction of BAT.

### Oxygen consumption determination

Mice were acclimated for 24 hours to an open-circuit indirect calorimeter system. The Comprehensive Lab Animal Monitoring System: Oxymax-CLAMS (Columbus Instruments, OH-USA) was calibrated as recommended by the manufacturer and used to measure the rate of O_2_ consumption (VO_2_), CO_2_ production (VCO_2_)_,_ respiratory exchange ratio (RER), heat rate (Kcal/h) and ambulatory activity during the light and dark periods. These data were acquired for 24 hours and were analyzed using Oxymax Windows software.

### Determination of IL6 levels

Mice were anesthetized, and blood was collected from the cava vein immediately after the last session of chronic exercise. Plasma was separated by centrifugation (1.100 × g) for 15 minutes at 4°C and stored at −80°C until the assay. IL-6 concentrations were determined using a commercially available *ELISA kit IL-6 Mouse* for mice (Invitrogen Life Tech KMC0062) following the manufacturer's instructions.

### Antibodies and chemicals

Nitrocellulose paper (Hybond ECL, 0.45 mm) was supplied by Amersham Pharmacia Biotech United Kingdom Ltd. (Buckinghamshire, United Kingdom). Ketamine was from Parke-Davis (São Paulo, SP, Brazil), diazepam and thiopental were from Cristália (Itapira, SP, Brazil). Anti-TGF-β (rabbit polyclonal, ab66043) was from Abcam plc. anti-phospho IκB-α (ser32) rabbit polyclonal SC7977-R), anti-UCP1 (goat polyclonal, M-17: SC-6529) antibodies were from Santa Cruz Biotechnology, Inc. anti-Tubulin (rabbit polyclonal, #2146), anti-β-actin (rabbit polyclonal, #4967) were from Cell Signaling Technology (Beverly, MA, USA). The antibody solution was 1:1000 for Western blots. Routine reagents were purchased from Sigma Chemical Co. (St. Louis, MO, USA).

### Protein analysis by immunoblotting

The hypothalamus and brown adipose tissue (BAT) were quickly removed and immediately frozen in liquid nitrogen. Then, the samples were minced coarsely and homogenized immediately as previously described [28]. The membranes were exposed to specific primary and secondary antibodies. After that, the membranes were exposed to SuperSignal^TM^ West Pico Chemiluminescent Substrate (Thermo Scientific), and the bands were analyzed by UN-SCAN-IT gel, 6.1. The whole mem-branes and the statistical analyses are presented in the Supplementary Figure 3.

### mRNA Isolation and Real-Time PCR

Total RNA content of brown adipose tissue (BAT) and hypothalamus was extracted using Rneasy® Mini Kit and QIAcube equipment by QIAGEN. The protocol was followed according to the recommendations of the manufacturer. In the exercised group, BAT was extracted after the last (20^th^) session of exercise. 2.0 mg of total RNA was reverse transcribed with High Capacity cDNA Kit (Applied Biosystems, Foster City, California, EUA). Real-time PCR was performed using 20ng of cDNA, 0.25ml of each primer, 3.0ml of TaqMan® Fast Advanced Master Mix (Applied Biosystems, Foster City, California, EUA) and RNase-free water to a total volume of 10ml. Data were analyzed based on the 2^^-dct^ method. Primers used for hypothalamus and Bat analyses were: *Tgfβ*:Mm03024053_m1; *Tnf*:Mm00443258_m1; *Il1-β*: Mm00434228_ml;*Il10*:Mm01288386_m1;*Tlr4*:Mm00445273_m1;*F4/80:;*Mm00802529_m1;*Npy*:Mm01410146_m1;*Ucp1*:Mm01244861_m1. Mouse GAPD (GAPDH) Endogenous Control (Catalog number: 4352339E).

### Statistical analysis

All statistical analyses were performed using the Student's t-test (comparisons between two groups) or one-way ANOVA (comparisons between more than two groups) with the Newman-Keuls Multiple Comparison test. For the Western blotting analysis, we used comparisons and quantified by optical densitometry (UN-SCAN-IT gel, 6.1). Significance was established at the mean ± SEM. *p*<0.05 level.

## SUPPLEMENTARY MATERIAL FIGURES


