# Supplementary material for: Chronic exercise reduces hypothalamic transforming growth factor-β1 in middle-aged obese mice
Source: Aging (Albany NY). 2017 Aug 28;9(8):1926–40. doi: 10.18632/aging.101281 (PMC5611986; doi:10.18632/aging.101281)
Supplement: Supplementary file 1 [file aging-09-1926-s001.pdf]

SUPPLEMENTAL MATERIAL

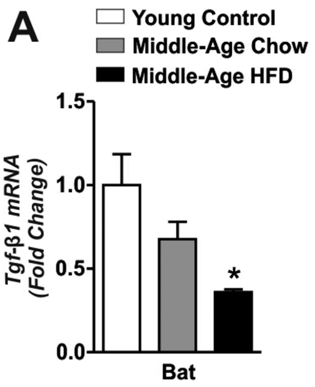

Figure S1. Effects of long-term of HFD on *Tgfβ-1 mRNA* level in the brown adipose tissue in Middle-Aged obese mice. Real time PCR assay shown *Tgfβ1* mRNA level in the brown adipose tissue of Y-Chow, MA-Chow and MA-HFD mice (n=4-7 per group). The animals kept 8 hours in fasting before of extraction of BAT. Data are expressed as ± SEM.*, *p*<0.05 vs. Young control group.

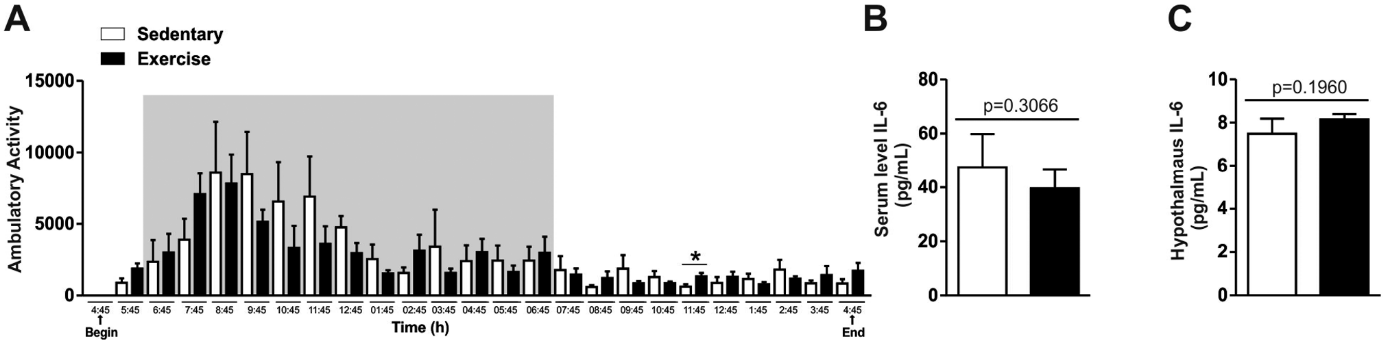

Figure S2. Ambulatory activity, serum and hypothalamic levels of IL-6 after chronic exercise protocol in Middle-Aged obese mice. Ambulatory activity analysis hour per hour (**A**) (n=4 per group). Serum level and hypothalamic of IL-6 (**B** and **C**) (n=5-6 per group). Data were expressed by using mean ± S.E.M. *, p<0.05, vs. Sedentary group.

BAT					
	UCP1	Tubulin			
	Pixel Total		Corrected to tubulin	Media (Control group)	Adjustmement to 100%
middle hfd 1	25028	544410	4.59727044	27.21872221	16.89010382
middle hfd 2	1330106	1875240	70.92990764		260.5923492
middle hfd 3	52924	863503	6.12898855	100	22.51754694
Middle hfd exe 4	291595	874810	33.33238075		122.4612254
middle hfd exe 5	951070	1287791	73.85282239		271.3309678
middle hfd exe 6	1226798	1138752	107.7317976		395.8003494
BAT					
	UCP1	Tubulin			
	Pixel Total		Corrected to tubulin	Media (Control group)	Adjustmement to 100%
middle hfd 1	9673	373651	2.588779369	13.96008246	18.54415528
middle hfd 2	38515	177182	21.73753542		155.7120846
middle hfd 3	160510	914382	17.5539326	100	125.7437601
Middle hfd exe 5	751235	1004434	74.79187284		535.7552367
middle hfd exe 6	974946	981971	99.28460209		364.7658451
middle hfd exe7	1053855	923633	114.0988899		419.1926757

Unpaired t test	
P value	0.0051
P value summary	**
Are means signif. different? (P < 0.05)	Yes
One- or two-tailed P value?	Two-tailed
t, df	t=3.573 df=10

Figure S3C. Statistical analysis of Figure 3H (UCP1 and Tubulin).

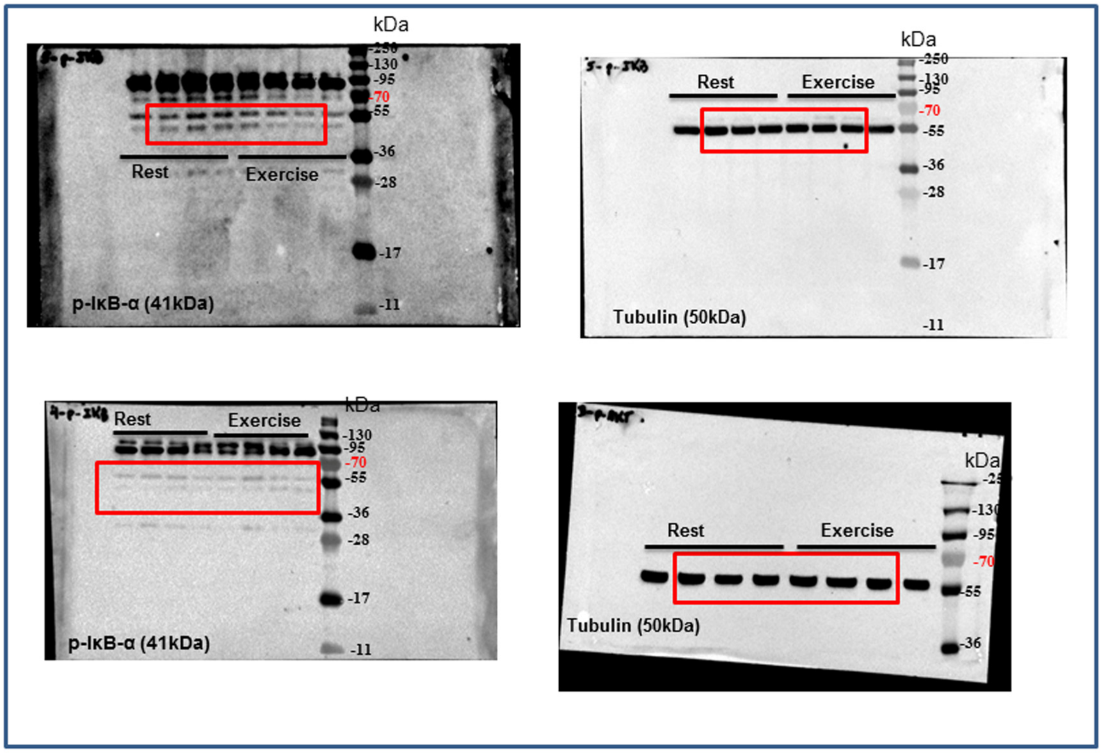

Figure S3D. Original western blot of Figure 4A (pIkB-alpha and Tubulin).

Hypothalamus					
	pIkB	Tubulin			
	Pixel Total		Corrected to tubulin	Media (Control group)	Adjustmement to 100%
middle hfd 1	141191	1190525	11.85955776	27.77100661	42.70481774
middle hfd 2	283907	1228524	23.10960144		83.21484981
middle hfd 3	443177	1148415	38.59031796	100	138.9590176
middle hfd 4	433532	1155329	37.52454928		135.1213149
middle hfd exe 5	332769	1133609	29.35483046		105.7031561
middle hfd exe 6	165925	1171526	14.16315131		50.99977652
middle hfd exe7	135865	1095899	12.39758408		44.64218477
middle hfd exe 8	167096	1131537	14.76717067		53.17477641

Hypothalamus					
	pIkB	Tubulin			
	Pixel Total		Corrected to tubulin	Media (Control group)	Adjustmement to 100%
middle hfd 1	48483	1979973	2.448669755	3.325508918	73.63293303
middle hfd 2	49507	1961019	2.524554836		75.91484185
middle hfd 3	87709	1783541	4.917689024	100	147.8777879
middle hfd 4	63951	1874779	3.411122058		102.5744372
middle hfd exe 5	63043	1943186	3.244311147		11.68236785
middle hfd exe 6	93462	1923951	4.857816025		17.49240167
middle hfd exe7	109481	1754254	6.240886439		22.47266917
middle hfd exe 8	113689	1715634	6.626646476		23.8617439

Unpaired t test	
P value	0.0040
P value summary	**
Are means signif. different? (P < 0.05)	Yes
One- or two-tailed P value?	Two-tailed
t, df	t=3.434 df=14

Figure S3E. Statistical analysis of Figure 4A (pIkB-alpha and Tubulin).

Horizontal Inverted (4blots)

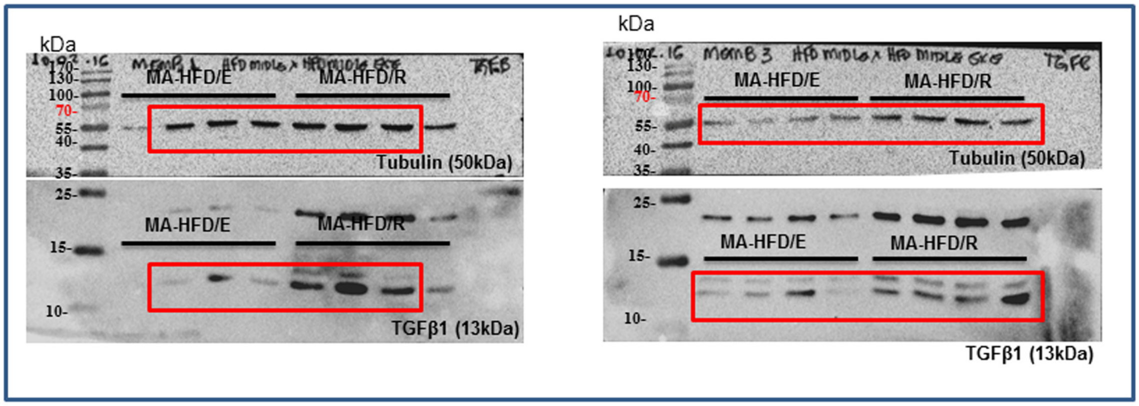

Figure S3F. Original western blot of Figure 4C (TGF beta 1 and Tubulin).

	TGFβ1	Tubulin			
	Pixel Total		Corrected to tubulin	Media (Control group)	Adjustmement to 100%
middle hfd 1	240060	557743	43.04132907	90.09602007	47.77273073
middle hfd 2	709176	829205	85.52480991		94.92629068
middle hfd 3	1166983	862110	135.3635847	100	150.2436895
middle hfd 4	804248	833812	96.45435662		107.057289
middle hfd exe 5	88517	661327	13.3847552		14.8561004
middle hfd exe 6	331066	709882	46.63676498		51.76340192
middle hfd exe7	31073	638579	4.865960202		5.400860325
middle hfd exe 8	2240	182052	1.230417683		1.365673735

Hypothalamus

	TGFβ1	Tubulin			
	Pixel Total		Corrected to tubulin	Media (Control group)	Adjustmement to 100%
middle hfd 1	689881	485560	142.0794547	68.87366206	206.2899669
middle hfd 2	325845	635455	51.27743113		74.45143702
middle hfd 3	279315	605603	46.12179926	100	66.96580069
middle hfd 4	189610	526461	36.0159632		52.29279541
middle hfd exe 5	305	381434	0.079961409		0.088751322
middle hfd exe 6	280494	325550	86.16003686		95.63134619
middle hfd exe7	37815	189243	19.98224505		22.17883213
middle hfd exe 8	14973	350144	4.276240632		4.746314686

Unpaired t test	
P value	0.0048
P value summary	**
Are means signif. different? (P < 0.05)	Yes
One- or two-tailed P value?	Two-tailed
t, df	t=3.351 df=14

Figure S3G. Statistical analysis of Figure 4C (TGF beta 1 and Tubulin).
